# Anti-HIV Activity and Immunomodulatory Properties of Fractionated Crude Extracts of *Alternaria alternata*

**DOI:** 10.3390/microorganisms12061150

**Published:** 2024-06-05

**Authors:** Mbali X. Kubheka, Sizwe I. Ndlovu, Nompumelelo P. Mkhwanazi

**Affiliations:** 1 HIV Pathogenesis Programme, Doris Duke Medical Research Institute, School of Laboratory Medicine and Medical Sciences, University of KwaZulu-Natal, Durban 4041, South Africa; 2 Department of Biotechnology and Food Technology, Faculty of Science, University of Johannesburg, Johannesburg 2092, South Africa; msizwe@uj.ac.za

**Keywords:** anti-HIV-1, immunomodulatory, *A. alternata*, endophytic fungi

## Abstract

Developing new anti-human immunodeficiency virus (HIV) drug candidates that target different sites in HIV-1 replication, with better resistance profiles and lower drug toxicity, is essential to eradicating HIV. This study investigated the potential of fractionated crude extracts of *Alternaria alternata* as immunomodulatory or anti-HIV drug candidates. Solid-phase extraction (SPE) was used to fractionate *A. alternata* PO4PR2 using three different columns: MAX (Mixed-mode, strong Anion-eXchange), MCX (Mixed-mode, strong Cation-eXchange), and HLB (Hydrophilic–Lipophilic Balance) with methanol gradient methods (5%, 45%, and 95%). An MTT (3-[4,5-dimethylthiazol-2-yl]-2,5 diphenyl tetrazolium bromide) assay was used to assess the cell viability and cytotoxicity of the fractionated crude extract *A. alternata* PO4PR2 in the TZM-bl cell lines. This was followed by a luciferase-based antiviral assay to assess the antiviral activity of *A. alternata* PO4PR2. A time of addition (TOA) assay was performed to ascertain the mechanism of inhibition employed by the fractionated crude extract of *A. alternata* PO4PR2 in the HIV life cycle. The p24 titer was determined using an ELISA, while a luciferase-based antiviral assay was used to evaluate the HIV percentage inhibition for different HIV-1 replication cycles. The TOA assay was established using antiviral drugs that target different sites in the HIV replication cycle. These included maraviroc, azidothymidine, raltegravir, and amprenavir. The immunomodulatory effect of the fractionated crude extracts on CD4+ T cells was measured by a flow cytometric analysis, for which fluorochrome-labelled monoclonal antibodies were used as markers for activation (CD38 and HLA-DR) and exhaustion (PD-1). The MCX fraction demonstrated a more significant anti-HIV inhibition than that of the fractions generated in other columns, with an IC_50_ of 0.3619 µg/mL, an HIV inhibition of 77%, 5% HLB (IC_50_: 0.7232 µg/mL; HIV inhibition of 64%), and 5% MAX (IC_50_: 5.240 µg/mL; HIV inhibition of 67%). It was evident from the time of addition data that the crude extract and the 5% MCX fraction inhibited viral binding (68%), reverse transcription (75%), integration (98%), and proteolysis (77%). It was shown that *A. alternata* (the MCX fraction) have a significant inhibitory effect on reverse transcription (75% HIV inhibition) and integration (100% HIV inhibition). The 5% MCX (*p* = 0.0062), 5% HLB (*p* = 0.0269), and 5% MAX (*p* = 0.0117) fractionated *A. alternata* crude extracts had low levels of CD4+ T cell (CD38 + HLA-DR+) activation compared to those of the AZT treatment, while CD4+ T cell activation was insignificant. The 5% MAX and HLB *A. alternata* fractions may possess immunomodulatory compounds with less anti-HIV-1 activity. *A. alternata* could be a key source of innovative anti-HIV drugs with immunomodulatory characteristics.

## 1. Introduction

Highly active antiretroviral (HAART) drugs have reduced the burden of the virus for people living with HIV (PLWH), with improved treatment outcomes and an increased life expectancy. Despite the apparent success of HAART therapy, there are several challenges present in the fight against HIV. Firstly, HIV-1 cannot be cured because antiretroviral treatment is not able to remove the virus from latently infected cells or viral reservoirs. Secondly, the prolonged use of highly active antiretroviral therapy has been linked to adverse events that compromise patients’ health, such as drug toxicity, including mitochondrial toxicity, hypersensitivity, lipodystrophy, dyslipidemia, type 2 diabetes, drug–drug interactions, and viral rebound upon treatment interruption [1,2,3]. In addition, the development of drug-resistant strains against anti-HIV-1 therapies has impeded the effectiveness of antiretroviral treatment outcomes. Therefore, the search for new antiretrovirals that are safe and can overcome current limitations continues.

Microbial natural products have high levels of structural diversity and bioactivity that cannot be rivalled by their various counterparts [4]. Medicinal plants are considered to be an interesting source of endophytes, with novel bioactive metabolites that have not been explored in previous phenotypic screening studies [5,6]. Endophytic fungi, for example, synthesise chemically complex compounds that confer a selective advantage to the plant host in natural environments [7]. Secondary metabolites produced by endophytic fungi have a high level of chemical and structural diversity, with a range of bioactive properties, including cytotoxic, immunosuppressive, antimicrobial, antimalarial, antiviral, and antioxidant [8,9,10,11].

Previous research has shown that the mangrove plant *Bruguiera gymnorrhiza* harboured a bacterial endophytic *Streptomyces* sp. that produced neomycin, which exhibits selective anti-HIV activity [12]. Xiamycin (with the geometric mean of its IC_50_ > 30 µM) inhibits R5 viruses. It does not affect HIV-1 infections that are X4 tropic [12,13]. Comparably, mulberroside C isolated from the bark of *Morus alba* and a fungal endophyte associated with this plant has been proven to be a potent and effective antiviral agent against HIV-1 and may even have protease activity [14]. Curcumin extracted from fungal endophytes of *Curcuma longa* L. was shown to be a potent antiviral agent, and its analogues were also effective in inhibiting HIV replication by targeting HIV protease [15], HIV integrase [16,17], and HIV-1 Tat protein [18]. According to previous research, Alterotoxin V from *Alternaria tenuissima* inhibits HIV-1 with an IC_50_ value of 0.9 µM [19]. Bashyal et al. (2014) [19] suggested that effective anti-HIV-1 therapy could be developed by manipulating the epoxy perylene structural framework in altertoxins. In addition, partially purified coumarins derived from fungal endophytes *Alternaria* spp., isolated from *Calophyllum inophyllum,* inhibited the activities of three viral enzymes: integrase (98%), reverse transcriptase (82.81%), and protease (78%) [20]. These studies highlight the potential of using secondary metabolites from *A. alternata* as a source for HIV-1 antagonist drug development.

Plant coumarins and their derivatives display anti-HIV activity through different mechanisms, including the inhibition of the HIV-1 reverse transcriptase and integrase and cellular components that regulate HIV-1 replication [21]. Furthermore, coumarins can be extracted from the various plant species’ roots, stems, bark, leaves, seeds, fruits, or flowers and have been found to have anti-HIV-1 properties [22]. Coumarins with anti-HIV-1 activity have been identified from *Calophyllum inophylum* and *Calphylum lanigerrum* plants. Calanolide A, also known as 11,12-dihydro-2H,6H,10H-dipyron[2,3-f2′,3′-h]chromen-2-one, was isolated from *Calophyllum lanigerum* and identified as the first anti-HIV-1 substance [23,24]. It has demonstrated a broad spectrum of anti-HIV-1 activity, including AZT and pyridinone drug-resistant strains [23]. Due to difficulties in isolating calanolide A from natural sources and its total synthesis, additional clinical trials have been halted, and its therapeutic index is low [24]. In light of this constraint, a recent work chemically synthesised calanolide A analogues by adding nitrogen heterocycles or aromatic groups into ring C [24]. This synthesised calanolide A analogue demonstrated moderate anti-HIV-1 activity on reverse transcriptase. Therefore, isolating endophytic fungi from *Calophylum* species that contain coumarin derivatives with anti-HIV-1 properties could solve the problem associated with the production of coumarins.

Bioactive secondary metabolites produced by endophytic fungi may directly possess anti-HIV-1 properties. They could target the virus directly, function as post factors, or exert immunomodulatory effects, meaning they might boost the immune system [25,26]. Previously, *Tinospora crispa* has been shown to have immunomodulatory effects by stimulating the production of IFN-γ, IL-6, and IL-8. In the *T. crispa* fraction, cordioside, quercetin, eicosenoic acid (paullinic acid), and boldine were detected by an LC-MS phytochemical analysis, which may be the cause of the plant’s immunostimulatory effects [27]. Furthermore, endophytic fungi have been reported as sources of HIV-1 protease inhibitors and possess immunomodulatory effects [28]. In addition, the secondary metabolites from an endophytic fungus isolated from *Ageratum conyzoide* showed antibacterial and immunomodulatory properties [29]. The study showed that at 100 and 200 mg/kg, the fungal extract significantly increased total white blood cell and neutrophil counts, indicating immunostimulatory activity. The evidence suggests that endophytic fungi produce secondary metabolites with antibacterial, antiviral, and immunomodulatory properties depending on the context and the target cells.

Nzimande et al. [30] isolated the endophytic fungi from *Hypoxis* spp., identified them as *A. alternata,* and further showed that these fungal isolates had anti-HIV-1 activity and were not cytotoxic in TZM-bl cells. A chemical analysis of the fractionated *A. alternata* crude extracts was conducted using gas chromatography–mass spectrophotometry and showed the presence of cyclotrisiloxane octamethyl (22.92%); propaninitrile (16,67%); pyrrolol[1,2-a]pyrazine-1,4-dione, hexahydro-3-(2-methyl propyl) (10.42%); silane, diethyl ethoxy (2-ethoxyethyloxy) (4.17%); coumarin, 3,4-dihydro-4,5,7-trimethyl-4,5,7-trimethyl-2-chromanone (13.7%); and 1,2-cyclobutanedicarbonitrile (2.08%) [30]. These chemical compounds were previously reported to possess biological properties, such as antiviral, antimicrobial, anti-inflammatory, and antioxidant activities (Table S1) [30,31,32,33]. However, the mechanism by which the crude extracts of *A. alternata* inhibit HIV-1 was not elucidated. This study aims to determine the mechanism of action of the fractionated crude extracts from *A. alternata* using a time of addition test. In this study, *A. alternata* crude extracts were first fractionated by solid-phase extraction using different columns: HLB, MAX, and MCX, as shown by Stoksck et al. [34]. The immunomodulatory properties of the fractionated crude extracts were also evaluated by measuring T cell exhaustion and activation.

## 2. Materials and Methods

### 2.1. Cell Culture

TZM-bl cells (NIH AIDS Research and Reference Reagent Programme; Washington, DC, USA) were produced from HeLa cells and modified to express CD4, CCR5, and CXCR4. They were also integrated with reporter genes from firefly luciferase. The TZM-bl and 293T/17 cells were grown in a monolayer in a sterile 75 cm^2^ culture flask using Dulbecco’s Modified Eagle Medium (DMEM) (Gibco, Thermo Fisher Scientific, Waltham, MA, USA) supplemented with 10% foetal bovine serum (FBS; heat inactivated and gamma irradiated) (LTC Biosciences, Gainesville, FL, USA), 25 mM HEPES (Gibco, Thermo Fisher Scientific, Waltham, MA, USA), and 50 µL/mL gentamicin (Gibco, Thermo Fisher Scientific, Waltham, MA, USA). It was incubated at 37 °C with 5% CO_2_. Confluent cells were rinsed with PBS (Gibco, Thermo Fisher Scientific, Waltham, MA, USA), trypsinised (0.25% Trypsin-EDTA, Gibco, Thermo Fisher Scientific, Waltham, MA, USA), counted using the 0.4% trypan blue (Invitrogen, Thermo Fisher Scientific, Waltham, MA, USA) method, and subcultured as needed. Trypan blue dye was adopted because non-viable cells absorb the dye while viable ones do not. In RPMI-1640 media (Gibco, Thermo Fisher Scientific, Waltham, MA, USA) supplemented with 10% (*v*/*v*) heat-inactivated foetal calf serum (FCS), the Jurkat cell line and peripheral blood mononuclear cells (PBMCs) were incubated at 37 °C under 5% CO_2_.

### 2.2. Secondary Metabolite Production

Fungal plugs (6 mm^2^) of *A. alternata* PO4PR2, isolated from *Hypoxis* species (voucher no. 18233 [30]) were sampled from a five-day-old malt agar plate and inoculated in 200 mL of malt extract broth in a one-litre Erlenmeyer flask (Rankem, Gurgaon, India). The fungal cultures were then incubated for 21 days at 30 °C under static conditions in the dark. The samples were then extracted with an equal volume of methanol (Sigma-Aldrich, Johannesburg, South Africa), followed by overnight shaking at 150 rpm using Rotory shaker (Reflecta Laboratory supplies, Glassware and Chemicals, Germiston, South Africa). All samples were then filtered with sterile gauze to remove mycelia and dried at 40 °C with a mild nitrogen gas steam [30,35]. Fungi-free cultures were included as controls and treated the same way as the experimental cultures to ensure that the observed activity resulted from fungal metabolites [36].

### 2.3. Fractionation of A. alternata PO4PR2 Using Mixed-Mode Solid-Phase Extraction

The solid-phase extraction of *A. alternata* PO4PR2 crude extracts was performed as described by Nzimande et al. [30] using a fractionation method derived from Stoszko et al. [34]. Briefly, one mL of crude extracts suspended in 50% high-performance liquid chromatography (HPLC)-grade methanol (MeOH, Sigma Aldrich, Johannesburg, South Africa) was spiked with 20 µL of phosphoric acid and loaded onto HLB (hydrophilic–lipophilic blender), MAX (mixed mode, strong anion exchange), and MCX (mixed mode, strong cation exchange) Oasis cartridges (Waters Corporation, Milford, MA, USA). Sample fractions were obtained by desalting the adsorbed chemicals and eluting them step-by-step with increasing methanol (MeOH) concentrations of 5%, 45%, and 95%. The collected fractions were dried using mild nitrogen stream at 40 °C. The samples were kept at −80 °C until further screening. Previously, the chemical analysis of fractionated *A. alternata* crude extracts was performed via gas chromatography–mass spectrophotometry [30].

### 2.4. Screening of the Cytotoxic Effects and Cell Viability of A. alternata PO4PR2 Using MTT (3-[4,5-Dimethylthiazol-2-yl]-2,5 Diphenyl Tetrazolium Bromide) Assay

The MTT viability assay assessed cell viability and cytotoxicity, following the manufacturer’s instructions (Thermo Fisher, Johannesburg, South Africa). A 96-well culture plate was seeded with 15,000 TZM-bl cells/200 µL DMEM/well and incubated at 37 °C with 5% CO_2_. Following that, in a separate 96-well plate (The Scientific Group, Maspeth, NY, USA), 25 µL of each crude extract, azidothymidine (AZT), and fractions from SPE (MAX, MCX, and HLB) were serially diluted in DMEM, containing 10% heat-inactivated foetal bovine serum, 50 μg/mL gentamycin, and 25 mM HEPES (LTC Biosciences, Gainesville, FL, USA) buffer. Azidothymidine (AZT) was included at a concentration of 300 µg/mL to serve as a positive control, and uninfected cells and DMEM alone were utilised as negative controls. The plates were incubated for 24 h at 37 °C under 5% CO_2_. After the incubation period, each well received 120 µL of MTT solution, comprising 100 µL fresh DMEM and 20 µL of MTT (Thermo Fisher, Johannesburg, South Africa) (5 mg/mL MTT salt in 0.1 M PBS). The supernatant (treatment medium) was then removed before the plate was incubated for 4 h at 37 °C with 5% CO_2_. After discarding the MTT solution, 100 µL of 0.2% DMSO (dimethyl sulfoxide) was added to each well, and the formazan crystals were allowed to dissolve for one hour at 37 °C in the incubator. The optical density was determined using a Victor Nivo multimode plate reader at 540 nm (PerkinElmer Inc., Waltham, MA, USA). The following formula was used to determine the percentage of cell viability based on the absorbance data:

(1) $$\left(\%\right)\;Cell\;Viability=\frac{\left(Sample\;absorbance-Cell-free\;sample\;blank\right)}{\left(Mean\;media\;control\;absorbance\right)}\times 100$$

GraphPad Prism version 5.01 (San Diego, CA, USA) was used to create a dose–response curve (concentration versus the percentage of cells that were viable in the samples) to determine the maximum cytotoxicity concentration at 50% (CC_50_).

### 2.5. Generation of Viruses (pNL4.3) by Transfection

To prepare the HIV-1 NL4.3 viruses, 293T cells (5 × 10^6^ cells in 12 mL growth medium in a T-75 culture flask) were transfected with 12 µg NL4.3 plasmid DNA (wild type) using a PolyFect transfection reagent, FuGENE 6 (Promega, Madison, WI, USA), in the growth medium [37]. After incubation for 30 min at room temperature (18–25 °C), the transfection complexes were introduced into 293T/17 cells in a T-75 culture flask and incubated for 48–72 h, with one media change for every 6 h of incubation. The flask contents were then filtered through a 0.45 µm filter to remove any remaining cell debris, and the virus-containing culture fluid was kept at −80 °C. The NL4.3 virus at the 50% tissue culture infective dose (TCID_50_) was prepared using a quadruplicate 5-fold dilution in a growth medium in 96-well culture plates (The Scientific Group, Maspeth, NY, USA). Freshly trypsinised TZM-bl cells (10,000 cells in volume of 100 µL) were introduced to each well with DMEM, containing an optimum concentration of DEAE-dextran (44 µg/mL) (Thermo Fisher, Johannesburg, South Africa). The culture plates were incubated for 48 h. After that, 100 µL of culture media was removed from each well and replaced with Bright-Glo reagent, as per the manufacturer’s protocol (Promega, Madison, WI, USA). Following this, the cells were allowed to lyse for two minutes at room temperature, and 150 µL of the cell lysate was transferred to a 96-well black solid plate (Costar, Berlin, Germany). The concentration resulting in 50,000 relative luminescence units (RLUs) was determined using a luciferase assay, and the reading was recorded in a Victor Nivo multimode microplate reader luminometer (Perkin-Elmer Life Sciences, Shelton, CT, USA).

### 2.6. Luciferase-Based Antiviral Assay

An antiviral assay based on luciferase was used to assess the HIV-1 inhibition of fungal extracts [30,38,39]. In short, 110 µL of DMEM supplemented with 10% heat-inactivated foetal bovine serum, 50 µg/mL gentamycin, and 25 mM HEPES buffer was serially diluted (10-fold) with 11 µL of each extract (or drug) sample. Then, 50 µL was serially diluted each time in 96-well flat-bottom culture plates (Whitehead Scientific, Cape Town, South Africa). For the fractions (HLB, MAX, and MCX) with 5%, 45%, and 95% methanol, the starting concentration ranged from 2 to 74 µg/mL. For the crude extract, it was 285 µg/mL, and for the positive drug control (AZT), it was 300 µg/mL. All TZM-bl cells, except for the cell control (negative control), were infected with the HIV-1 NL4.3 virus TCID_50_ (50 µL/well). After that, the plate was covered and incubated at 37 °C with 5% CO_2_ for one hour. About ten to fifteen minutes before being used, TZM-bl cells were suspended at a density of one million cells per millilitre in D-MEM supplemented with 44 µg/mL of DEAE dextran (Thermo Fisher, Johannesburg, South Africa). Next, a 100 µL cell solution containing 10,000 cells was added to each well. After that, the plates were covered and incubated at 37 °C with 5% CO_2_ for 48 h. Next, 150 µL of culture was removed from each well and replaced with 100 µL of Bright-Glo luciferase reagent (Promega, Madison, WI, USA). The plates were incubated for two minutes at room temperature in the dark to allow for total cell lysis. After that, 150 µL was moved to a matching 96-well black plate (Costar, Berlin, Germany). A Victor Nivo multimode microplate reader (PerkinElmer, Waltham, MA, USA) was used to read the data. The percentage inhibition was estimated by calculating the difference in average number of RLUs between the test wells (cells + drug + virus) and the cell control wells (cells only, column 1) and dividing the results by the difference in average number of RLUs for virus control. GraphPad Prism Software (v.5.00.288) (San Diego, CA, USA) was used to determine the half-maximal inhibitory concentration (IC_50_).

### 2.7. Determining the Mode of Action of the Fractionated A. alternata Crude Extracts and Their Inhibitory Mechanism in the HIV-1 Life Cycle Using Time of Addition Assay

#### 2.7.1. HIV-1 p24 Time-Based ELISA to Measure the p24 Titre

The interference of the anti-HIV compounds with the HIV replication cycle was identified by a time of addition (TOA) assay. The time of addition experiment was carried out as described by Daelemans et al. [40] with minor adjustments. Briefly, a 24-well culture plate containing 50,000 Jurkat cells/500 µL/well was seeded with the HIV-1 NL4.3 virus TCID50 (100 µL/well) and incubated at 37 °C with 5% CO_2_. Antiretroviral drugs, including maraviroc (CCR5 antagonist, 0–1 h), azidothymidine (nucleoside reverse transcriptase inhibitor, 3–8 h), raltegravir (integrase inhibitor, 10–12 h), and amprenavir (protease inhibitor, 16–24 h), were added. With various time intervals according to the drugs’ mechanism of action during the HIV-1 replication cycle, a training set was established. The experimental setup included *A. alternata* PO4PR2 crude extract or 5% MCX fraction (25 µL/well) added to the infected cells. The inhibition patterns of the crude extract or the 5% MCX fraction were observed compared to those established with the ARV drug training set. The HIV-1 p24 titre in the culture supernatant was determined using the Quicktiter Lentiviral Quantification kit (Cell Biolabs Inc., San Diego, CA, USA), following the manufacturer’s instructions. The p24 level was determined at 480 nm using the multimode Victor Nivo microplate reader (PerkinElmer, Waltham, MA, USA). The viral titre was calculated as per the manufacturer’s instructions as follows:

Calculate viral titre:

The average genome size of lentivirus is eight kbp; therefore, the following can be calculated:

1 ng lentiviral RNA = (1 × 10^−9^) g/(8000 bp × 660 g/bp) × 6 × 1023 = 1.1 × 10^8^ VP

$$Virus\;titre\;(VP/mL)=\frac{Amount\;of\;lentiviral\;RNA\;(ng)\times 1.1\times 108\;VP\times (20\;µL/5\;µL)}{Viral\;sample\;volume\;(mL)}$$

$$Virus\;titre\;(VP/mL)=\frac{Amount\;of\;lentiviral\;RNA\;(ng)\times 4.4\times 108\;VP/ng}{Viral\;sample\;volume\;(mL)}$$

#### 2.7.2. Luciferase-Based Time of Addition Assay to Determine the Percentage Inhibition

With minor adjustments, the luciferase-based time of addition assay was carried out in accordance with Lara et al. [41]. Briefly, 10,000 TZM-bl cells/150 µL/well were seeded in a 96-well culture plate (incubated at 37 °C, 5% CO_2_), and the cells were then infected with 50 µL of HIV-1 NL4.3 virus TCID50. During the HIV-1 replication cycle, ARV controls, a crude extract, and the 5% MCX fraction were individually added to infected cells (15 µL/well) at varied intervals as described in the ELISA-based time of addition assay. The plate was then incubated for 48 h at 37 °C with 5% CO_2_. Following a 48 h period, 150 µL of culture was removed from each well and replaced with 100 µL of Bright-Glo luciferase reagent (Promega, Madison, WI, USA). The plates were incubated for two minutes at room temperature in the dark to allow for total cell lysis. After that, 150 µL of culture and Bright-Glo luciferase reagent were transferred to a 96-well black plate (Costar, Berlin, Germany). A Victor Nivo multimode microplate reader (PerkinElmer, Waltham, MA, USA) was used to read the data. The average RLU difference between the test wells (cells + drug + virus) and the cell control wells (cells only, column 1) was calculated, and the results were divided by the difference in average number of RLUs for virus control to estimate the percentage inhibition.

### 2.8. Effect of the Fractionated Crude Extracts from A. alternata P04PR2 on CD4^+^ and CD8^+^ T Cell Activation and Exhaustion

#### 2.8.1. Flow Cytometry Staining

Peripheral blood mononuclear cells (PBMCs) harvested from HIV-1-negative people (FRESH cohort: BREC REF: BF131/11) and stored in liquid nitrogen were used in this study. The cryopreserved PBMCs were thawed by aliquoting eight millilitres of pre-warmed R10 (RPMI, 10% FBS, 1% glutamine, 1% Pen Strep, 1% HEPES) into a 15-millilitre falcon tube in a 37 °C water bath until the pellet was half-thawed. The thawed PBMC cells were centrifuged at 1500 rpm for 10 min at room temperature after being pre-warmed in 8 mL of R10 media. The supernatant was discarded, 10 millilitres of R10 was added, and the mixture was centrifuged for 10 min at 1500 rpm at room temperature. The cell pellet was resuspended in two mL of R10 (for roughly 10 million cells) and allowed to rest in 5% CO_2_ at 37 °C for at least two hours with the falcon tube cover loosened. The crude extracts and fractions were added to five HIV-1 (NL4.3)-infected PBMCs and five uninfected PBMCs. AZT was included as a positive control, and untreated PMBCs from HIV-1-negative participants were included. Furthermore, 20 µL of *A. alternata* crude extract and each of 5% MAX, 5% HLB, and 5% MCX fractions were added to a suspension of PBMC that had been prepared at a density of 1 × 10^5^ cells/well in R10 in a 24-well culture plate. After that, 1500 µL of the cell suspension and 100 µL of NL4.3 (TCID50) were added to each well, except the negative control well (which contained uninfected PBMC). The percentage of T cells expressing the markers of exhaustion (PD-1) and activation (HLA-DR and CD38) was assessed in fresh peripheral blood mononuclear cells [42,43]. The following fluorochrome-labelled monoclonal antibodies (MAbs) were incubated with fresh PBMC samples for 30 min: CD3-BV650 (BD), CD4-APC (eBioscience, San Diego, CA, USA), CD8-FITC or CD56-BV510, CD45RO APC, HLA-DR PEcF, and CD38 BV711 (BD bioscience, Piscataway, NJ, USA). A FACS CANTO II (BD) was used for data acquisition. Compensation was carried out using FlowJo software (version 9.5.3; Tree Star Inc., Ashland, OR, USA) using antibody capture beads (BDs) stained individually with each antibody employed in the test samples.

#### 2.8.2. Statistical Analysis

Data were analysed using GraphPad Prism V5.0 software (GraphPad Software Inc., San Diego, CA, USA). Dose–response curves were generated to determine the IC_50_s or CC_50_s (GraphPad Software, CA’s version 9.5.3, Tree Star Inc., Ashland, OR, USA). FlowJo software was used to analyse the flow cytometry data. GraphPad Prism V5.0 software was used to generate the graphs using non-parametric testing (Mann–Whitney test) and the unpaired *t*-test.

## 3. Results

### 3.1. Cytotoxicity and Cell Viability of the Fractionated A. alternata Using MTT Assay

**Figure 1:**
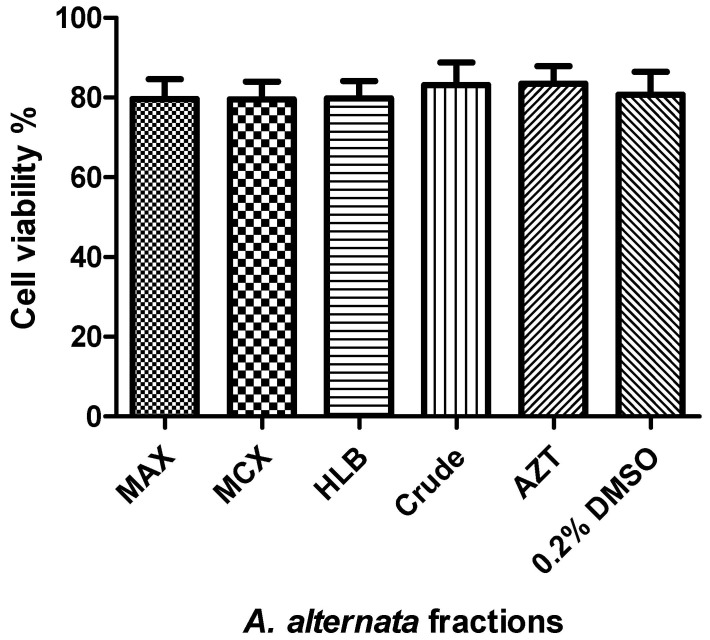
*A. alternata* PO4PR2 fractions MAX, MCX, HLB, and crude extract. In addition, AZT and 0.2% DMSO were tested for cell viability. The cell viability percentage is displayed on the y-axis, while the bioactive fraction containing crude extract, AZT, and solvent DMSO is represented on the x-axis.

Figure 1 shows that the viability of the crude extract, fractions, 0.2% DMSO (the solvent), and AZT (the positive drug control) remained over 80%.

### 3.2. The Anti-HIV-1 Effects of Fractionated A. alternata PO4PR2 Crude Extract and Fractionated Fractions (MAX, MCX, HLB) Using Luciferase-Based Antiviral Assay

**Figure 2:**
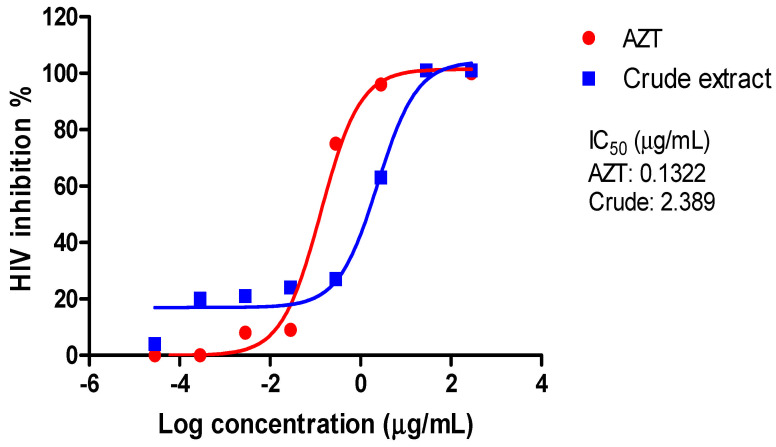
HIV-1 percentage inhibition curve of AZT and *A. alternata* crude extracts PO4PR2.

**Figure 3:**
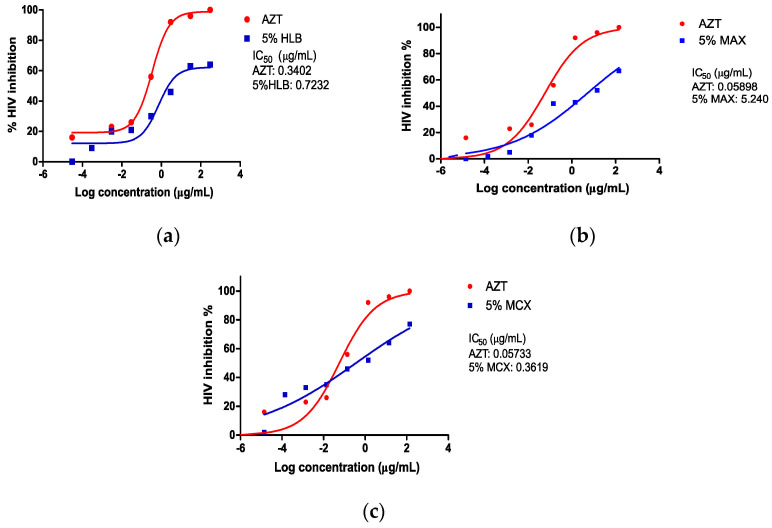
HIV-1 percentage inhibition of fractionated crude extract of *A. alternata* PO4PR2. (**a**) 5% HLB fraction; (**b**) 5% MAX fractions; and (**c**) 5% MCX fractions.

A luciferase-based antiviral assay was used to assess the viral inhibition of the *A. alternata* PO4PR2 crude extracts and fractionated crude extracts on the TZM-bl cell line. There was a 100% HIV-1 inhibition in the presence of AZT (nucleotide reverse transcription inhibition) and the crude extract of *A. alternata* PO4PR2 (Figure 2). The AZT’s inhibitory concentration (IC_50_) was 0.1322 µg/mL and the crude extract’s was 0.017 µg/mL. The inhibition observed in the solvent only (0.2% DMSO) was 17%, with an IC_50_ of 26.2647 µg/mL. Table 1 and Figure 3 show the HIV-1 inhibition (%) in relation to the fractions. The antiviral activity was also examined for the three fractions: MAX, MCX, and HLB (5%, 45%, and 95% methanol).

The anti-HIV-1 efficacy of the HLB fractions decreased, as the methanol gradient increased with a 64% HIV-1 inhibition for the 5% methanol fraction, 48% HIV-1 inhibition for a 45% methanol fraction, and 32% HIV-1 inhibition for a 95% methanol fraction (Table 1). The same pattern was also observed with the MAX fractions, in which there was a 67% HIV-1 inhibition after treatment with a 5% methanol fraction, 63% HIV-1 inhibition with a 45% methanol fraction, and 40% HIV-1 inhibition with a 95% methanol fraction. The MCX fractions followed a similar pattern: a 77% HIV-1 inhibition was observed when treated with a 5% methanol fraction, 74% HIV-1 inhibition after treatment with a 45% methanol fraction, and 53% HIV-1 inhibition after treatment with a 95% methanol fraction. The potent activity was calculated using the selective index (SI) for the AZT, crude extract, and 5% MeOH (MAX, MCX, and HLB) fractions. The selective index values were 6.74, 172.17, 113, 2559, and 7874 for the 5% MeOH (MAX, MCX, and HLB) fractions, crude extract, and AZT, respectively. Lastly, the antiviral activity of the crude extract and MCX fraction (which showed a good level of antiviral activity) was evaluated against all positive drug controls that were utilised for the time of addition assay to determine the inhibitor concentration at which the binding (or response) is halved (IC_50_) (S1) (Table 2). The MCX fraction was chosen because of its high level of suppression of HIV-1 in the TZM-bl cell line.

### 3.3. The Mode of Action of the Fractionated Crude Extracts and Their Tentative Inhibitory Mechanism in the HIV-1 Life Cycle Using Time of Addition Assay

**Figure 4:**
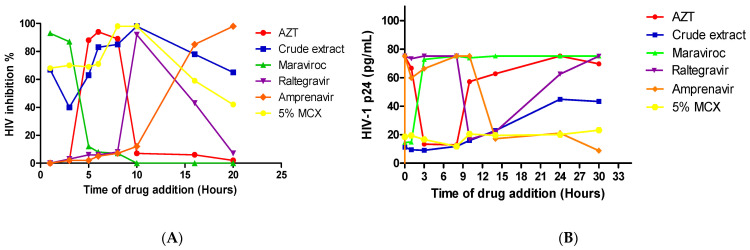
Luciferase-based time of addition assay for HIV replication cycle intervention in fractionated *A. alternata* PO4PR2 fractions. (**A**) Cell lines infected with HIV-1 NL4.3. The x-axis displays the number of hours since the crude extracts and fractions were added, while the y-axis displays the percentage of HIV inhibition. (**B**). Using HIV-1 p24 ELISA, the x-axis displays the amount of time in hours that the crude extracts and fractions were added, and the y-axis displays the concentration of HIV-1 p24 (pg/mL).

The time of addition assay was conducted using the luciferase time-based assay (Figure 4A) and the ELISA p24 time-based test (Figure 4B) to determine the active fractions’ inhibitory mechanisms and mode of action in the HIV-1 life cycle. Since the 5% MCX partially purified fraction showed a greater level of antiviral activity against HIV-1 than the other fractions, a time of addition experiment was carried out to identify the inhibitory target of the *A. alternata* crude extract and 5% MCX fraction. Jurkat cell lines were infected with NL4.3 and treated with the crude extract and 5% MCX fraction at various intervals to quantify them using the p24 Quicktiter Lentiviral Quantification kit (Cell Biolabs Inc., San Diego, CA, USA). One hour after HIV-1 entry, the crude extract demonstrated a high level of HIV-1 inhibition; it was able to lower the HIV-1 p24 titre from 75.187 pg/mL (the virus control) to 11.22 pg/mL in comparison to the positive control, 14.64 pg/mL maraviroc. *A. alternata* crude extracts showed HIV-1 inhibition during reverse transcription (3–8 h) with the 16.69 pg/mL HIV-1 p24 titre. The inhibition effect of the crude extract was also observed during the integration stage, at which 11.69 pg/mL raltegravir was compared to 16.79 pg/mL raltegravir. The HIV-1 p24 titre was inhibited by a 5% MCX fraction during HIV-1 entry (0–2 h) (18.53 pg/mL), reverse transcription (3–8 h) to 9.06 pg/mL, and integration (8–12 h) (Figure 4B). There was a continuous decrease in the HIV-1 p24 titre even after integration. In addition, the luciferase time-based assay revealed that the 5% MCX fraction exhibited higher levels of anti-HIV activity, e.g., 5% MCX fractions (83%), than those of the crude extract (67%) (Figure 4A) during the virus’ entry and reverse transcription, with the level of HIV-1 inhibition ranging from 68% to 98% between 0 and 8 h (HIV-1 entry, reverse transcription, and integration).

The 5% MCX fraction demonstrated potent HIV-1 inhibition as evidenced by a 3.8-fold reduction in the viral p24 concentration from 75.187 pg/mL (virus control) to 19.61 (pg/mL) in comparison to the crude extract (22.89 pg/mL) during HIV-1 integration (10–12 h) and to the HIV-1 p24 titre (20.13 and 43.33 pg/mL). The TZM-bl cell line, on the other hand, showed that during virus entry (68%) and reverse transcription (98%), the 5% MCX fraction had more significant anti-HIV activity than during crude extract viral entry (67%) and reverse transcription (83%). The 5% MCX fraction showed 59% HIV inhibition during proteolysis, unlike the crude extract, which exhibited 64% HIV inhibition after proteolysis. Following integration, the anti-HIV activity of the crude extract and the 5% MCX fraction showed the same HIV-1 inhibition potential of 98% (Figure 4A).

### 3.4. Evaluation of the Effects of Fractionated Crude Extracts from A. alternata on CD4^+^ and CD8^+^ T Cell Activation and Exhaustion

#### Flow Cytometry Staining

**Figure 5:**
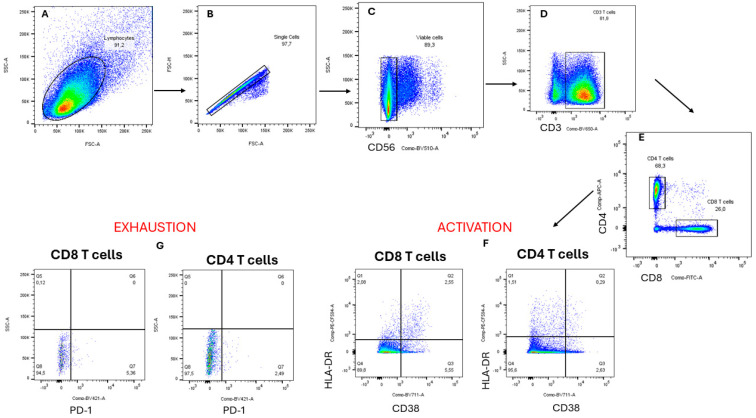
The T cell subpopulation analysis gating strategy: (**A**) The lymphocytes were gated according to the forward and side scatter areas; (**B**) The single cells from the lymphocytes were gated according to the forward scatter height and side scatter areas; (**C**) After that, the viable cells were gated according to the side scatter areas and the CD56+ T cell population (BV510-A); (**D**) After that, the CD3+ T cell population was gated according to the side scatter areas and the CD3+ T cell population (BV650); (**E**) From the CD3+ T cell population, the CD4+ T cell population and the CD8+ T cell population were gated according to the CD4+ T cells (APCs) and the CD8+ T cells (FITCs); (**F**) Finally, from the CD4 T cell population and the CD8+ T cell population, we evaluated the activation based on HLA-DR (PE-CF) and CD38 (BCV711); (**G**) We evaluated the exhaustion of CD4 T cell population and CD8 T cell population based on side scatter area and PD-1.

The potential immunomodulatory effects of the crude extract and partially purified *A. alternata* PO4PR2 fractions (MCX, HLB, and MAX) with antiviral activity were assessed using flow cytometry staining to measure T cell activation and exhaustion (Figure 5). AZT was used as a positive control, while crude extract and partially purified fractions (MCX, HLB, and MAX) were used to treat infected and uninfected PBMCs. AZT is one of the primary drugs used in HIV treatment, and research has previously shown that it is more sensitive to the inhibitory effects of AZT-naïve cytotoxic T cells than activated antigen-primed cells [43]. Activation (CD38 + HLA-DR) and exhaustion (PD-1) markers were detected in the PBMCs using fluorochrome-labelled monoclonal antibodies.

**Figure 6:**
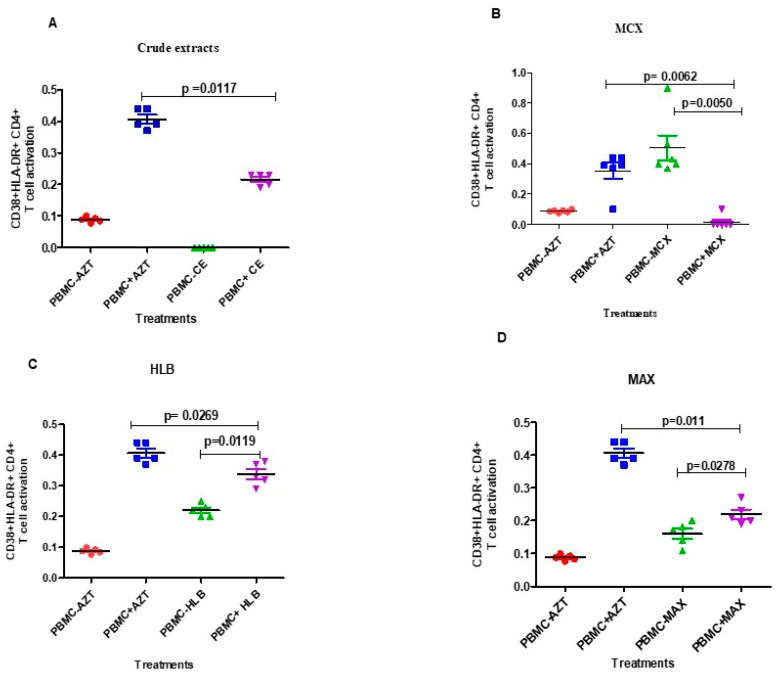
The *A. alternata* PO4PR2 fractions (MCX, HLB, and MAX) were tested for CD4+ T cell activation on HIV-1-infected PBMCs using flow cytometry. The x-axis displays the cell treated with different fractions, where PBMCs- are uninfected PBMCs and PBMCs+ are HIV-1-infected PBMCs, while the y-axis displays (%CD4 T cells + CD38 + HLA-DR) T cell activation. Azidothymidine (AZT) was included as a positive drug control. (**A**) Crude extract. (**B**) MCX. (**C**) HLB. (**D**) MAX. The red dot represent PBMC-AZT, blue square (PBMC + AZT), green (PBMC-crude extract), purple (PBMC + crude extracts).

Treatment with the crude extract resulted in low levels of CD38 + HLA-DR + CD4+ T cell activation, as shown in Figure 6, while treatment with the fractions (5%MCX, HLB, and MAX) and AZT resulted in T cell activation. In the HIV-1-infected PBMCs with MAX (*p*-value = 0.0278; Figure 6D) and HLB fractions (*p*-value = 0.0019; Figure 6C), there was a substantial increase in CD38 + HLA-DR + CD4+ T cell activation upon treatment with the fractionated *A. alternata* crude extracts in the 5% MAX column. When the fractionated *A. alternata* crude extract fraction (5% MCX) was applied to the HIV-1-infected PBMCs, there was a significant decrease in CD38 + HLA-DR + CD4+ T cell activation (*p*-value = 0.0050; Figure 6B). In both treated and untreated as well as infected and uninfected PBMCs, there was no evidence of CD8+ T cell activation.

**Figure 7:**
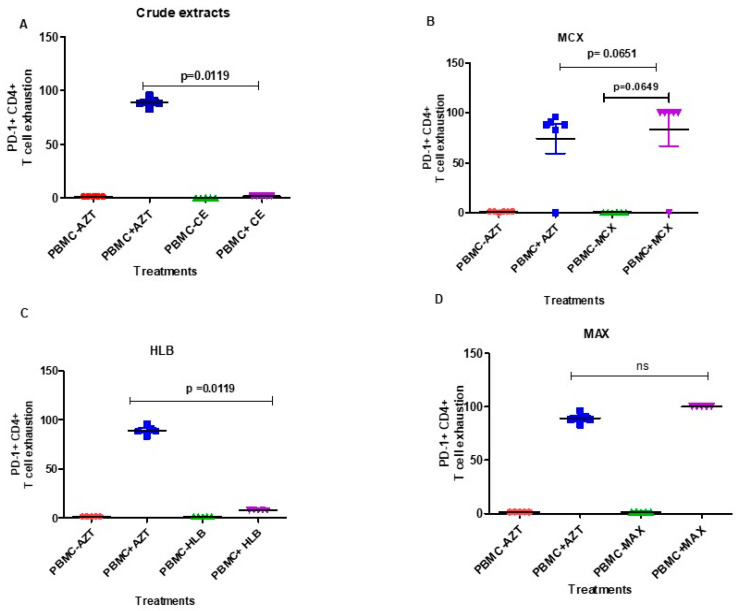
The CD4+ T cell exhaustion of the crude and *A. alternata* PO4PR2 fractions (MCX, HLB, and MAX) was determined for PBMCs using flow cytometry. The y-axis shows (%CD4 T cell + PD-1) T cell exhaustion, and the x-axis indicates the treatments, where uninfected PBMCs are labelled PBMCs- and infected PBMCs are labelled PBMCs+. Azidothymidine (AZT) was used as a positive drug control. (**A**) The crude extract; (**B**) The MCX fraction; (**C**) The HLB fraction; (**D**) The MAX fraction. The red dot represent PBMC-AZT, blue square (PBMC + AZT), green (PBMC-crude extract), purple (PBMC + crude extracts).

**Figure 8:**
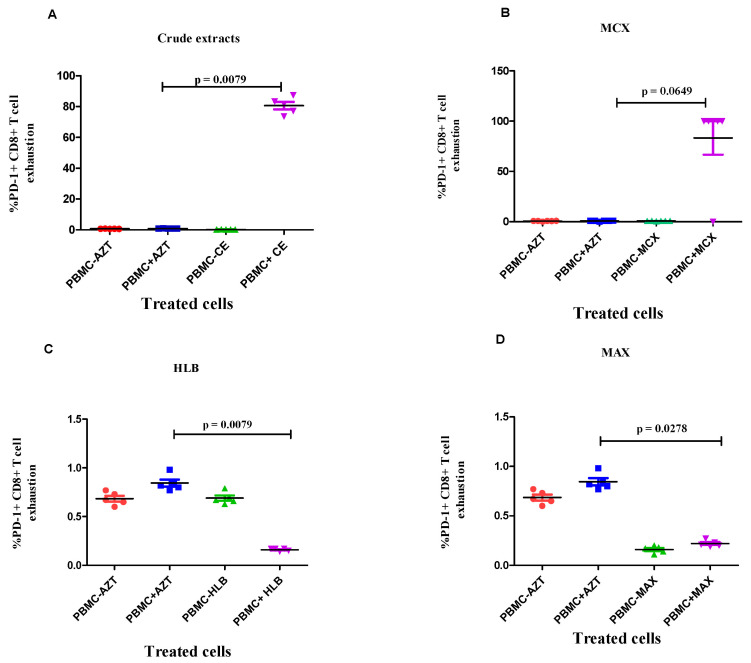
The CD8^+^ T cell exhaustion of the crude extracts and fractionated *A. alternata* PO4PR2 fractions (MCX, HLB, and MAX) was determined on PBMCs using flow cytometry. The y-axis shows (%CD8 + PD-1) T cell exhaustion, and the x-axis indicates the treatments, where uninfected PBMCs are labelled PBMCs- and infected PBMCs are labelled PBMCs+. Azidothymidine (AZT) was used as a positive drug control. (**A**) *A. alternata* crude extract; (**B**) 5% MCX fraction; (**C**) 5% HLB fraction; (**D**) 5% MAX fraction. The red dot represent PBMC-AZT, blue square (PBMC + AZT), green (PBMC-crude extract), purple (PBMC + crude extracts).

Compared to AZT, there was no distinct change in the PD-1 + CD4+ T cell exhaustion caused by the infected PBMCs after treatment with crude extract (Figure 7A). However, the level of PD-1 + CD8+ T cell exhaustion was significantly higher in the HIV-1-infected PBMCs treated with the *A. alternata* crude extract than in the HIV-negative PBMCs (*p*-value = 0.0112; Figure 8A). Additionally, PD-1 + CD4+ and PD-1 + CD8+ T cell exhaustion did not significantly increase in the HIV-1-infected PBMCs treated with the *A. alternata* fractionated crude extracts of 5% MCX (Figure 7B and Figure 8B; *p*-value = 0.0649). The fractionated crude extracts of 5% HLB in the HIV-1-infected PBMCs led to a significant increase in PD-1 + CD4+ T cell exhaustion (*p*-value = 0.0119), as seen in Figure 7C. In the HIV-1-infected PBMCs treated with the fractionated *A. alternata* of 5% HLB, there was a substantial decrease in PD-1+CD8+ T cell exhaustion compared to HIV-1-negative PBMCs (*p*-value = 0.0079; Figure 8C). The number of AZT-treated PBMCs remained high. When comparing the HIV-1-infected PBMCs treated with the MAX fraction to the HIV-1-negative PBMCs, there was no distinct increase in PD-1 + CD4+ T cell exhaustion (Figure 7D) or PD-1 + CD8+ T cell exhaustion (*p*-value = 0.278; Figure 8D).

## 4. Discussion

An endophytic fungus, *A. alternata,* isolated from *Hypoxis* species, showed strong anti-HIV-1 properties [30]. This study used a solid-phase extraction method to fractionate crude extracts from *A. alternata,* using different MAX, MCX, and HLB matrixes. The fractionated crude extracts showed significant levels of anti-HIV activity and were not toxic to the cells. Both the p24 ELISA and the luciferase-based time of addition assay demonstrated strong anti-HIV-1 activity in the MCX fraction of *A. alternata* during HIV-1 entry, reverse transcription, and integration. These findings imply that the bioactive compounds in the fractionated *A. alternata* crude extracts can act as immune stimulators and inhibitors of the virus at multiple stages of HIV-1 replication.

The luciferase antiviral-based assay assessed the anti-HIV-1 activity of *A. alternata* crude extract (PO4PR2) and fractionated crude extracts. Our findings indicated the presence of anti-HIV activity in the crude extract and fractionated crude extract of *A. alternata*, indicating that endophytic fungi are promising sources of antiretroviral drugs. The chemical fingerprints of these bioactive compounds can be elucidated in a metabolomic analysis using NMR and liquid chromatography–mass spectrophotometry. High-performance liquid chromatography (HPLC) and Fourier transform ion cyclotron resonance (FTICR) mass spectrometry were employed by Stosckz et al. [34] to offer a thorough analysis of latency-reversing drugs. The capacity to inhibit HIV-1 was not lost by the fractionated fractions (MCX, MAX, HLB) and was generated using increasing methanol gradients (5%, 45%, and 95%, respectively). However, the percentage inhibition decreased (Figure 3 and Figures S1–S3). These findings imply that the anti-HIV activity was reduced when the crude extract used several cartridges with a methanol gradient. However, the 5% methanol MCX fraction maintained a high level of anti-HIV-1 activity.

After a GC-MS analysis, Nzimande et al. [30] identified several putative bioactive compounds in the MCX fraction. These included propargylamine and 1,2-cyclobutanedicarbonitrile, as well as coumarin (3,4-dihydro-4,5,7-trimethyl-2-chromanone), which have been previously shown to have antiviral activity. The selective index of the 5% methanol MCX fraction was higher and exceeded 10, indicating possible therapeutic compounds that could be used in preclinical studies [44,45]. Of all the fractions studied, the MCX fraction exhibited the most significant selective index and the most effective HIV-1 inhibition, implying that the MCX fraction is safer and more effective than the other two tested fractions (Table 2). Subsequent investigations can assess the MCX fraction separately and identify the bioactive compound.

The time at which the *A. alternata* crude extracts and 5% MCX fraction inhibit HIV replication was putatively determined using a time of addition (TOA) assay. A “time of addition” is used to identify where and when in the HIV replication cycle the *A. alternata* crude extract and partially purified fraction interfere with HIV replication [46,47]. Our findings show that the HIV-1-infected cells treated with the 5% MCX fraction and crude extracts have low levels of HIV-1 p24 titre during entry and reverse transcription. When HIV-1-infected TZM-bl cells were treated with a 5% MCX fraction, however, they demonstrated potent HIV-1 inhibition (%) during entry, reverse transcription, and integration. This suggests that the *A. alternata* crude extract and 5% MCX fraction may possess the secondary metabolites that target the host cell receptors CD4, CCR5, or CXCR4 during entry or the viral envelope glycoproteins gp120 and gp41.

Compared to the crude extract, which exhibits potent HIV-1 inhibition in TZM-bl cell lines, the 5% MCX fraction exhibited a high level of HIV-1 inhibition during proteolysis and integration in the Jurkat cell line. Using the TZM-bl cell line, the crude extract and 5% MCX fraction exhibit the same level of HIV-1 inhibition during integration (98–100%). The bioactive chemicals present may also impede HIV replication and the infection of new cells. In conclusion, it has been demonstrated that the MCX fraction and crude extract from *A. alternata* block HIV-1 during its entrance and function as integrase, protease, and reverse transcriptase inhibitors. These findings corroborate the research of previous studies, which found that three viral enzymes—integrase (98%), reverse transcriptase (82.81%), and protease (78%)—showed HIV-1 potent inhibition when used with partially purified coumarins isolated from *Alternaria* sp. [20,48].

The HIV-1-infected PBMCs’ T cell activation and exhaustion were evaluated to determine the immunomodulatory effects of fractionated *A. alternata* secondary metabolites. ARVs can lower an HIV-1-positive person’s viral load and immunological activation level, which may assist in maintaining or improving T cell function and avoiding or reversing T cell exhaustion [49]. In this study, we observed that when partially purified fractions were added to the HIV-1-infected PBMCs, there was an increase in CD4+ T cell activation (CD38 + HLA-DR+). These cytokines function as messengers and promote CD4+ T cell activation and proliferation [49]. As a result, following an HIV infection, we noticed an increase in CD4+ T cell activation in the PBMCs. This increased CD4+ T cell activation in the PBMCs following HIV infection indicates that while the immune system is trying to combat the virus, more CD4+ T cells are being lost.

In comparison to the AZT-treated cells, the fraction (5% MCX, HLB, and MAX) treated cells and crude extracts of *A. alternata* reduced the activation of CD4+ T cells (CD38 + HLA-DR+) (Figure 6). After treatment with crude extracts and fractions, there may potentially be a decrease in the activation of CD4+ T cells on HIV-1-infected peripheral blood monoclonal cells (PBMCs). This could indicate that the treatment is effective in preventing HIV replication and activating CD4+ T cells, as evidenced by the reduced levels of activation markers, such as HLA-DR and CD38, on the CD4+ T cells in the infected PBMCs [48]. The MCX fraction demonstrated the lowest level of CD4+ T cell (CD38 + HLA-DR+) activation compared to that of the other tested compounds/extracts. Our results validate the use of the 5% MCX fraction of *A. alternata*, which is linked to the minimal activation of CD4+ T cells and may benefit the immune system. Other variables, such as CD4 T cell count and viral load, should also be considered as they might not be a reliable indicator of the drug’s safety or efficacy.

In this investigation, we also evaluated the activation of CD8+ T lymphocytes by *A. alternata*, crude extract, and fractionated crude extracts. Our findings showed that CD8+ T cell activation was either absent or low in the treated, untreated, infected, and uninfected PBMCs. This could indicate that CD8+ T cell activation is unnecessary because the antiviral drugs under evaluation efficiently inhibit the virus [50]. Nevertheless, further characterisation of the fractionated *A. alternata* crude extracts may yield more information, as the mechanism behind the lack of activation of CD8+ T cells remains unclear.

While T cell exhaustion was initially found in CD8 T cells, it is now recognised that CD4 T cells can also experience it, which results in a decreased production of IL-2, IFN-γ, and TNF-α as well as decreased support from CD4 T cells [51,52]. Our findings demonstrated that the level of infected PBMCs’ PD-1+CD4+ T cell exhaustion was reduced by the crude extracts and the 5% HLB fraction. In addition, PD-1 + CD4+ T cells treated with MCX and MAX fractions showed significant levels of exhaustion simultaneously (Figure 7). The loss of effector activities, such as cytokine generation, and increased expression of inhibitory receptors, such as PD-1, LAG-3, and TIM-3, which block T cell activation, indicate high levels of T cell exhaustion [53]. Compared to the MCX fraction and the crude extract, we found that the HLB and MAX fractions had lower levels of PD-1+CD8+ T cell exhaustion (Figure 8). This suggests that the bioactive compounds in the HLB and MAX fractions effectively prevent CD8+ T cell exhaustion, which helps maintain the immune system’s capacity to fight off the virus.

## 5. Conclusions

The presence of secondary metabolites in the MCX fraction (acidic compounds) that alter CD8+ T cells’ primary functions, such as the secretion of cytokines like IFN-g, IL2, granzyme, and perforin, is indicated by the high level of CD8+ T cell exhaustion found [53]. To identify the bioactive compounds that have anti-HIV-1 activity in 5% MCX fractions, further studies to identify and characterise these compounds are recommended. According to our findings, secondary metabolites may have mildly antiviral and immunomodulatory properties in crude extract, MAX, and HLB fractions. It is still necessary to investigate how these immunomodulators reduce T cell exhaustion in CD4+ T and CD8+ T cells. We therefore conclude that the fungal metabolites in *A. alternata* may possess compounds with both anti-HIV-1 and immunomodulatory properties. Further characterisation of these compounds using high-resolution liquid chromatography and nuclear magnetic resonance is recommended.

## Figures and Tables

**Table 1:** HIV-1 inhibition (%) and the IC_50_s of all *A. alternata* fractionated fractions on TZM-bl cells *.

*A. alternata* Fractions	IC_50_ (µg/mL)	% HIV Inhibition at the Maximum Concentration)
5% HLB	0.7232	64
45% HLB	0.1130	48
95% HLB	0.0338	32
5% MAX	5.240	67
45% MAX	0.03843	63
95% MAX	0.03313	40
5% MCX	0.3619	77
45% MCX	6.750	74
95% MCX	0.0110	53

* Eluted in 5%, 45%, and 95% methanol.

**Table 2:** Cell cytotoxicity concentrations (CC_50_s) and HIV inhibition concentrations (IC_50_s) on TZM-bl cells of the fractionated crude extracts of *A. alternata* PO4PR2.

Crude Extract	CC_50_ (µg/mL)	IC_50_ (µg/mL)	Selective Index (CC_50_/IC_50_)
5% MAX	35.31	5.240	6.74
5% MCX	62.31	0.3619	172.17
5% HLB	81.81	0.7232	113
Crude extract	43.5	0.017	2559
AZT	1041	0.1322	7874

## Data Availability

The raw data supporting the conclusions of this article will be made available by the authors on request.

## Supplementary Materials

The following supporting information can be downloaded at https://www.mdpi.com/article/10.3390/microorganisms12061150/s1, Figure S1: The HIV-1 percentage inhibition curves of *Alternaria alternata* crude extract and HLB fractions (neutral compounds) were tested with luciferase-based antiviral assay using TZM-bl cell lines. The TZM-bl cells were infected with HIV-1 (NL4.3) wild type and treated with the serial dilution of *Alternaria alternata* HLB fractions, crude extract with AZT as positive control and incubated for 48 h at 37 °C and 5% CO_2_. The y-axis represents the percentage of HIV inhibition, and the x-axis shows the log concentration in µg/mL. The red line represents the positive drug control AZT, and the blue line represents the fractions. The 0% HLB and 5% HLB fractions show high anti-HIV-1 (%) after SPE with an IC_50_ of 1.942 and 0.7232 (µg/mL) compared to 45% and 95% fraction with an IC_50_ of 0.1130 and 0.0338 (µg/mL). Figure S2: The HIV-1 percentage inhibition curves of *Alternaria alternata* MAX fractions (acidic compounds) were tested with luciferase-based antiviral assay using TZM-bl cell lines. The TZM-bl cells were infected with HIV-1 (NL4.3) wild type and treated with the serial dilution of *Alternaria alternata* MAX fractions with AZT as positive control and incubated for 48 h at 37 °C and 5% CO_2_. The y-axis represents the percentage of HIV inhibition, and the x-axis shows the log concentration in µg/mL. The red line represents the positive drug control AZT, and the blue line represents the fractions. The 5% MAX fraction shows high anti-HIV-1 (%) with an IC_50_ of 5.240 (µg/mL) compared to 0%, 45% and 95% fraction with an IC_50_ of 0.1172, 0.03843 and 0.03313 (µg/mL). Figure S3: The HIV-1 percentage inhibition curves of *Alternaria alternata* MCX fractions (basic compounds) were tested with luciferase-based antiviral assay using TZM-bl cell lines. The TZM-bl cells were infected with HIV-1 (NL4.3) wild type and treated with the serial dilution of *Alternaria alternata* MCX fractions with AZT as positive control and incubated for 48 h at 37 °C and 5% CO_2_. The y-axis represents the percentage of HIV inhibition, and the x-axis shows the log concentration in µg/mL. The red line represents the positive drug control AZT, and the blue line represents the fractions. The 0% MCX, 5% MCX and 45% MCX fractions show high anti-HIV-1 (%) after SPE with an IC_50_ of 0.03262, 0.3619 and 6.750 (µg/mL) compared to 95% MCX fraction with an IC_50_ of 0.01103 (µg/mL). Figure S4: The HIV-1 percentage inhibition curves of the positive drug controls were tested with luciferase-based antiviral assay using TZM-bl cell lines. The TZM-bl cells were infected with HIV-1 (NL4.3) wild type and treated with the serial dilution of positive controls (AZT, Maraviroc, Raltegravir and Amprenavir) and incubated for 48 h at 37 °C and 5% CO_2_. The y-axis represents the percentage of HIV inhibition, and the x-axis shows the log concentration in µg/mL. The red line represents the positive drug controls, and the blue line represents the crude extract. Maraviroc showed inhibition of 93% with an IC_50_ of 0.001684 (µg/mL), Zidovudine was 100% with an IC_50_ of 2.0550 (µg/mL), Raltegravir was 87% with an IC_50_ of 0.008331 (µg/mL), Amprenavir was 98% with an IC_50_ of 0.00168 (µg/mL), and the crude extract was 101% with an IC_50_ of 34.38 (µg/mL). Table S1: Chemical components of the *A. alternata* crude extracts analysed by Gas-chromatography-mass spectrophotometry [30].
