# Supplementary material for: Anti-HIV Activity and Immunomodulatory Properties of Fractionated Crude Extracts of Alternaria alternata
Source: Microorganisms. 2024 Jun 5;12(6):1150. doi: 10.3390/microorganisms12061150 (PMC11205553; doi:10.3390/microorganisms12061150)
Supplement: Supplementary file 1 [file microorganisms-12-01150-s001.zip › microorganisms-3000558-supplementary.pdf]

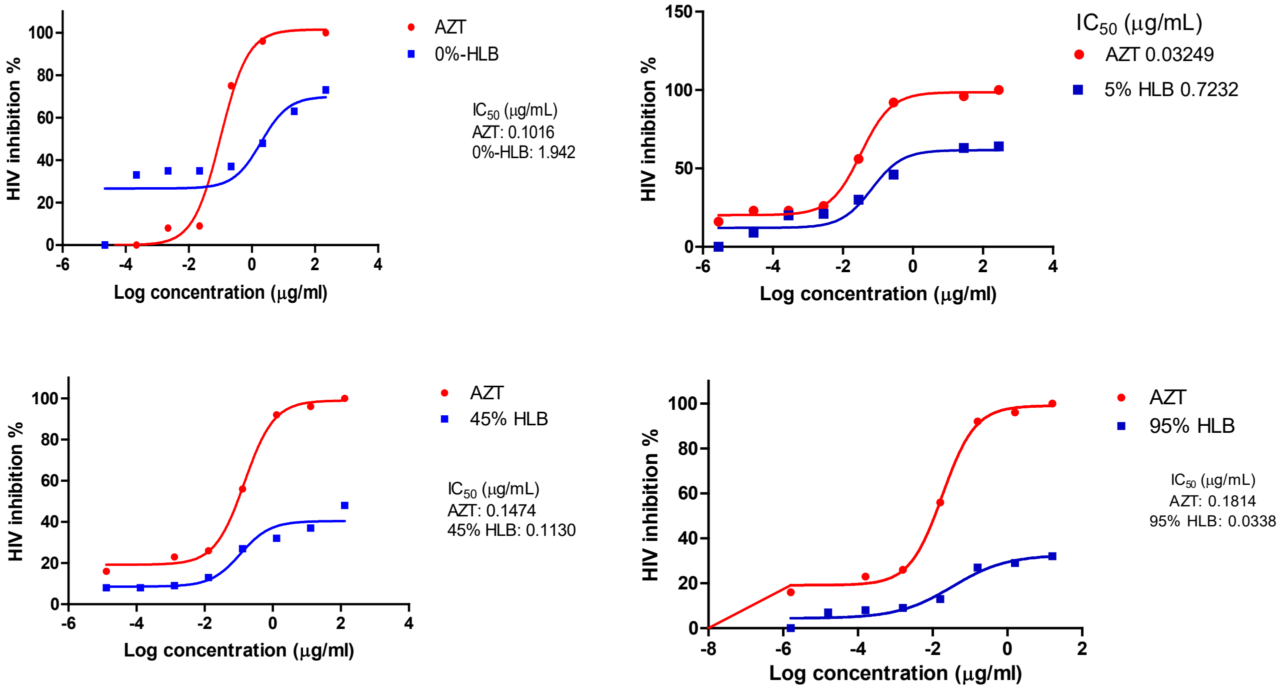

Figure S 1: The HIV-1 percentage inhibition curves of *Alternaria alternata* crude extract and HLB fractions (neutral compounds) were tested with Luciferase-based antiviral assay using TZM-bl cell lines. The TZM-bl cells were infected with HIV-1 (NL4.3) wild type and treated with the serial dilution of *Alternaria alternata* HLB fractions, crude extract with AZT as positive control and incubated for 48 h at 37°C and 5% CO_2. The y-axis represents the percentage of HIV inhibition, and the x-axis shows the log concentration in μg/mL. The red line represents the positive drug control AZT, and the blue line represents the fractions. The 0% HLB and 5% HLB fractions show high anti-HIV-1 (%) after SPE with an IC_{50} of 1.942 and 0.7232(μg/mL) compared to 45% and 95% fraction with an IC_{50} of 0.1130 and 0.0338 (μg/mL).

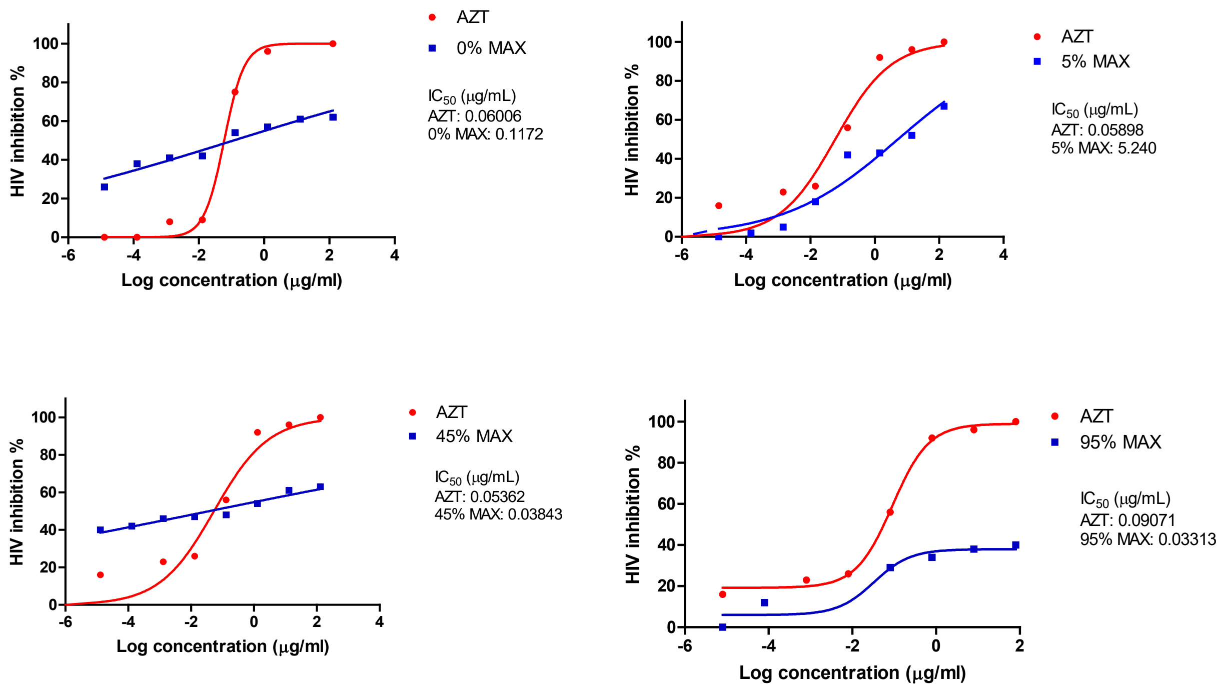

Figure S 2: The HIV-1 percentage inhibition curves of *Alternaria alternata* MAX fractions (acidic compounds) were tested with Luciferase-based antiviral assay using TZM-bl cell lines. The TZM-bl cells were infected with HIV-1 (NL4.3) wild type and treated with the serial dilution of *Alternaria alternata* MAX fractions with AZT as positive control and incubated for 48 h at 37°C and 5% CO_2. The y-axis represents the percentage of HIV inhibition, and the x-axis shows the log concentration in μg/mL. The red line represents the positive drug control AZT, and the blue line represents the fractions. The 5% MAX fraction shows high anti-HIV-1 (%) with an IC_{50} of 5.240 (μg/mL) compared to 0%, 45% and 95% fraction with an IC_{50} of 0.1172, 0.03843 and 0.03313 (μg/mL).

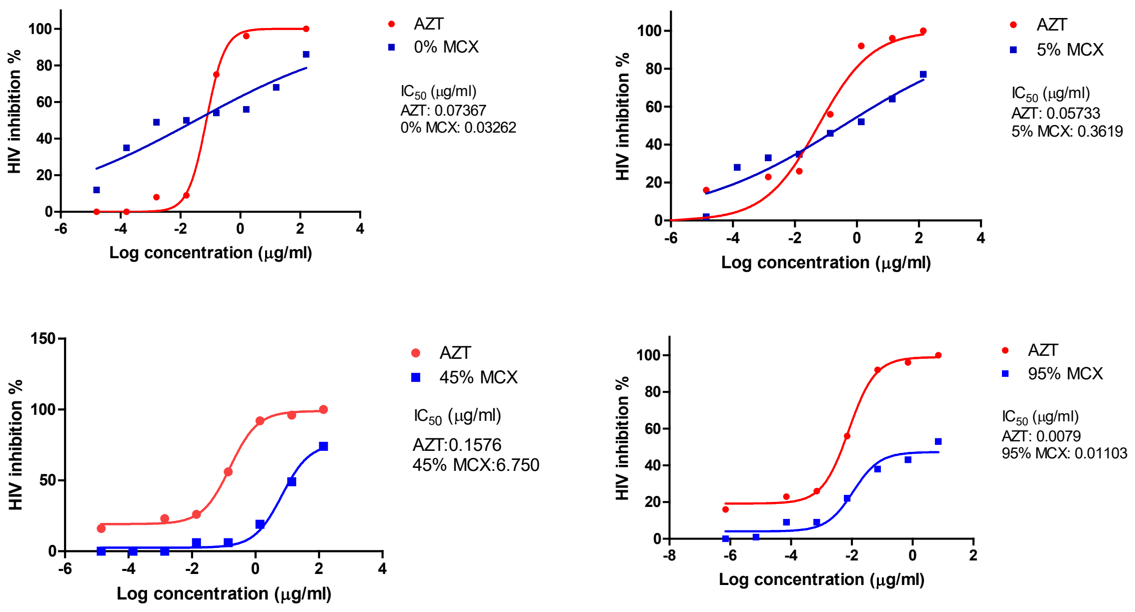

Figure S 3: The HIV-1 percentage inhibition curves of *Alternaria alternata* MCX fractions (basic compounds) were tested with Luciferase-based antiviral assay using TZM-bl cell lines. The TZM-bl cells were infected with HIV-1 (NL4.3) wild type and treated with the serial dilution of *Alternaria alternata* MCX fractions with AZT as positive control and incubated for 48 h at 37°C and 5% CO_2. The y-axis represents the percentage of HIV inhibition, and the x-axis shows the log concentration in µg/mL. The red line represents the positive drug control AZT, and the blue line represents the fractions. The 0% MCX, 5% MCX and 45% MCX fractions show high anti-HIV-1 (%) after SPE with an IC_{50} of 0.03262, 0.3619 and 6.750(µg/mL) compared to 95% MCX fraction with an IC_{50} of 0.011103 (µg/mL).

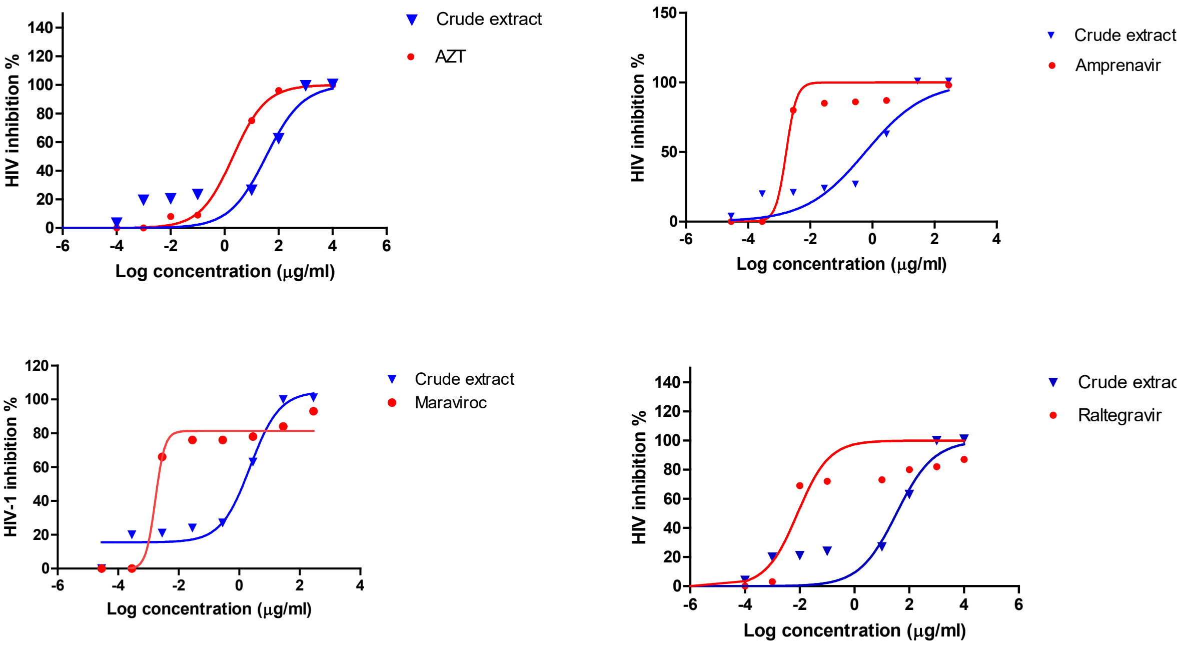

Figure S 4: The HIV-1 percentage inhibition curves of the positive drug controls were tested with Luciferase-based antiviral assay using TZM-bl cell lines. The TZM-bl cells were infected with HIV-1 (NL4.3) wild type and treated with the serial dilution of positive controls (AZT, Maraviroc, Raltegravir and Amprenavir) and incubated for 48 h at 37°C and 5% CO2. The y-axis represents the percentage of HIV inhibition, and the x-axis shows the log concentration in μg/mL. The red line represents the positive drug controls, and the blue line represents the crude extract. Maraviroc showed inhibition of 93% with an IC_{50} of 0.001684 (μg/mL), Zidovudine was 100% with an IC_{50} of 2.0550 (μg/mL), Raltegravir was 87% with an IC_{50} of 0.008331 (μg/mL), Amprenavir was 98% with an IC_{50} of 0.00168 (μg/mL), and the crude extract was 101% with an IC_{50} of 34.38 (μg/mL).

Supplementary Table 1 S: Chemical components of the *A.alternata* crude extracts analysed by Gas-chromatography-mass spectrophotometry [30]

Peak No.	Formula	IUPAC name	SI	Natural sources
2	$C_9H_{20}OSi$	1-Methyl-1-n-pentyloxy-1-silacyclobutane	71	*Macrocystis pyrifera and Waltheria indica*
4	$C_7H_{14}O_6$	alpha.-Methyl-D-mannopyranoside	74	*Gnetum gnemon*
6	$C_7H_{12}O$	6-Methyl-cyclohex-2-en-1-ol	62	*Arum hygrophilum*
7	$C_{10}H_{16}O$	Tricyclo[4.4.0.0(2,8)]decan-4-ol	39	*Mentha viridis*
8	$C_7H_{10}N_2O_2$	Pyrrolo[1,2-a]pyrazine-1,4-dione, hexahydro-	92	*Bacillus tequilensis MSI45*
11	$C_{17}H_{24}O_3$	7,9-Di-tert-butyl-1-oxaspiro(4,5)deca-6,9-diene-2,8-dione	69	*Cyathea Nilgirensis Holttum and Cordia sebestena*
8	$C_6H_{12}O_2$	Silane, butoxy trimethyl-	90	*Torilis arvensis*
9	$C_5H_6O_2$	2(3H)-Furanone, 5-methyl	87	*Tamarindus indica*
10	$C_9H_{22}O_3Si$	Silane, diethyl ethoxy(2-ethoxyethyloxy)-	66	*Alternaria alternata*
11	$C_5H_4O_3$	2,5-Furandione, 3-methyl-	69	*Curculigo orchiodes and Couroupita guianensis*
12	$C_8H_{24}O_4Si_4$	Cyclotetrasiloxane, octamethyl-	89	*Olea europaea*
13	$C_6H_6O_3$	Levoglucosenone	92	*Aspergillus sojae*
14	$C_9H_{10}O$	Benzaldehyde, 2,4-dimethyl-	92	*Hypericum triquetrifolium and Ganoderma lucidum*
15	$C_{14}H_{22}$	Benzene, 1,3-bis(1,1-dimethylethyl)-	67	*Pila virens*
16	$C_{17}H_{22}F_2O_4$	Oxalic acid, 3,5-difluorophenyl nonyl ester	65	*Trigonella foenumgraecum*
22	$C_{11}H_{14}O_2$	1,3-Dioxolane, 4,5-dimethyl-2-phenyl-	58	*Punica granatum*
24	$C_{18}H_{34}O_4$	Oxalic acid, bis(6-ethyl oct-3-yl) ester	74	*Cullenia exalliarata and Centella asiatica*
25	$C_{14}H_{16}O_5$	4-O-Methyl-2,3-O-benzal-d-mannosan	47	*Eucommia ulmoides (Wood vinegar production and refining)*
26	$C_{14}H_{28}O_2$	Hexanoic acid, octyl ester	58	*Neisseria gonorrhea and Hibiscus sabdariffa*
27	$C_{16}H_{34}O_5Si_2$	Disiloxane, 1,1,3,3-tetramethyl-1,3-bis[3-(oxiranylmethoxy)propyl]-	48	*Boswellia sacra*
29	$C_7H_{14}S$	4-Ethylthiane	49	*Allium sativum*
30	$C_{11}H_{18}O_2S$	4-Allyl-2-t-butyl-4-methyl-1,3-oxathiolan-5-one	54	*Aconitum Heterophyllum*
32	$C_{16}H_{24}O$	Allyl ionone 4	51	*Allium cepa*

35	$C_{10}H_{14}N_2O_3$	3-Methyl-1,4-diazabicyclo[4.3.0]nonan-2,5-dione, N-acetyl	83	*Bacillus amyloliquefaciens*
37	$C_{22}H_{40}$	Cyclopentane, 1,1'-[3-(2-cyclopentyl methyl)-1,5-pentanediyl]bis-	50	*Mentha haplocalyx and Phellinus pomaceous*
41	$C_{22}H_{42}O_2$	Phytol, acetate	91	*Cassia siamea and Celtis occidentalis*
42	$C_{20}H_{40}O$	3,7,11,15-Tetramethyl-2-hexadecen-1-ol	91	*Adiantum capillus-veneris and Ulva lactuca*
44	$C_{14}H_{22}N_2O_2$	5,10-Diethoxy-2,3,7,8-tetrahydro-1H,6H-dipyrrolo[1,2-a:1',2'-d]pyrazine	74	*Urtica dioica and Urtica urens and Bacillus safensis*
49	$C_{38}H_{68}O_8$	l-(+)-Ascorbic acid 2,6-dihexadecanoate	75	*Brassica juncea*
50	$C_8H_{20}OSi$	Silane, ethoxytriethyl-	81	*Enhalus acoroides and Streptomyces lavendulae*
3	$C_{17}H_{40}O_5Si_2$	3,7,11,14,18-Pentaoxa-2,19-disilaeicosane, 2,2,19,19-tetramethyl-	71	*Plantago ovata*
4	$C_{12}H_{19}NO_6$	3,5-Di-O-acetyl-2,4-di-O-methyl-6-deoxy-d-gluconitrile	41	*Methylomonas sp.*
6	$C_{10}H_{14}N_2O_3$	3-Methyl-1,4-diazabicyclo[4.3.0]nonan-2,5-dione, N-acetyl	80	*Bacillus amyloliquefaciens and Amanita subglobosa*
12	$C_9H_{27}AsO_3Si_3$	Arsenous acid, tris(trimethylsilyl) ester	72	*Gloriosa superba and Gymnopilus junonius*
2	$C_8H_5N_3O_4$	2,3-Dihydroxy-6-nitroquinoxaline	56	*Ficus carica*
3	$C_8H_{24}O_4Si_4$	Cyclotetrasiloxane, octamethyl-	95	*Olea europaea and Trichoderma reesei*
4, 5	$C_{10}H_{30}O_5Si_5$	Cyclopentasiloxane, decamethyl	86	*Olea europaea and Nigrospora sphaerica*
6	$C_{24}H_{50}O_4Si_2$	Dodecanedioic acid, bis(tert-butyldimethylsilyl) ester	59	*Mallotus oppositifolius*
13	$C_{10}H_{18}S$	3-Cyclohexylthiolane	38	*Brevibacillus brevis*
14	$C_{12}H_{20}N_2O$	2-Isopropyl-octahydrobenzo[e][1,2]oxazine-3-carbonitrile	46	*Illicium verum*
15	$C_6H_{18}O_3Si_3$	Cyclotrisiloxane, hexamethyl-	76	*Paecilomyces lilacinus and Dillenia scabrella*
16	$C_8H_{24}O_4Si_4$	Cyclotetrasiloxane, octamethyl-	95	*- repeat*
9	$C_{20}H_40O$	3,7,11,15-Tetramethyl-2-hexadecen-1-ol	66	*Annona muricata*

10	$C_{17}H_{37}BO_6Si_2$	beta.-D-Galactopyranoside, methyl 2,3-bis-O-(trimethylsilyl)-, cyclic butyl boronate	36	*Calotropis procera*
13	$C_{19}H_{19}BrO_2$	(E)-2-bromobutyloxychalcone	62	*Streptomyces avermitilis and Ziziphus spina*
15	$C_{15}H_{13}N$	1-Methyl-3-phenylindole	59	*Hunteria umbellata*
7, 12	$C_{12}H_{14}ClN$	Pyridine, 1,2,3,6-tetrahydro-1-methyl-4-[4-chlorophenyl]-	56	*Auricularia auricula-judae and Ocimum Gratissmum*
10	$C_{12}H_{16}O_3$	Asarone	38	*Acorus calamus (Acorus ifrom south africa)*
2	$C_{16}H_{48}O_8Si_8$	Cyclooctasiloxane,	43	*Solanum nigrum*
4	C_4H_6	1,2-Butadiene	84	*Aspergillus clavatonanicus strain MJ31*
5	$C_{14}H_{22}O_2$	1,2-Benzenediol, 3,5-bis(1,1-dimethylethyl)-	64	*Ziziphus abyssinica*
6	$C_{13}H_{20}N_2SSi$	1,2-Benzisothiazol-3-amine tbdms	60	*Thevetia neriifolia*
9	$C_{12}H_{14}O_2$	Coumarin, 3,4-dihydro-4,5,7-trimethyl--2-chromanone	53	*Pulicaria incisa*
